# A new haematocytometric index: Predicting severity and mortality risk value in COVID-19 patients

**DOI:** 10.1371/journal.pone.0254073

**Published:** 2021-08-05

**Authors:** Meltem Kilercik, Özlem Demirelce, Muhittin Abdulkadir Serdar, Parvana Mikailova, Mustafa Serteser

**Affiliations:** 1 Acibadem Labmed Clinical Laboratories, İstanbul, Turkey; 2 Department of Medical Biochemistry, AcibademMehmet Ali Aydınlar University, İstanbul, Turkey; Azienda Ospedaliero Universitaria Careggi, ITALY

## Abstract

**Introduction:**

Coronavirus disease 2019 (COVID-19) caused by SARS-CoV-2 virus, is a major public health concern spanning from healthy carriers to patients with life-threatening conditions. Although most of COVID-19 patients have mild-to-moderate clinical symptoms, some patients have severe pneumonia leading to death. Therefore, the early prediction of disease prognosis and severity is crucial in COVID-19 patients. The main objective of this study is to evaluate the haemocytometric parameters and identify severity score associated with SARS-CoV-2 infection.

**Methods:**

Clinical and laboratory records were retrospectively reviewed from 97 cases of COVID-19 admitted to hospitals in Istanbul, Turkey. The patient groups were subdivided into three major groups: Group 1 (Non-critical): 59 patients, Group 2 (Critical-Survivors): 23 patients and Group 3 (Critical-Non-survivors):15 patients. These data was tested for correlation, including with derived haemocytometric parameters. The blood analyses were performed the Sysmex XN-series automated hematology analyser using standard laboratory protocols. All statistical testing was undertaken using Analyse-it software.

**Results:**

97 patients with COVID-19 disease and 935 sequential complete blood count (CBC-Diff) measurements (days 0–30) were included in the final analyses. Multivariate analysis demonstrated that red cell distribution width (RDW) (>13.7), neutrophil to lymphocyte ratio (NLR) (4.4), Hemoglobin (Hgb) (<11.4 gr/dL) and monocyte to neutrophil ratio (MNR) (0.084) had the highest area under curve (AUC) values, respectively in discrimination critical patients than non-critical patients. In determining Group 3, MNR (<0.095), NLR (>5.2), Plateletcount (PLT) (>142 x10^3^/L) and RDW (>14) were important haemocytometric parameters, and the mortality risk value created by their combination had the highest AUC value (AUC = 0.911, 95% CI, 0886–0.931). Trend analysis of CBC-Diff parameters over 30 days of hospitalization, NLR on day 2, MNR on day 4, RDW on day 6 and PLT on day 7 of admission were found to be the best time related parameters in discrimination non-critical (mild-moderate) patient group from critical (severe and non-survivor) patient group.

**Conclusion:**

NLR is a strong predictor for the prognosis for severe COVID-19 patients when the cut-off chosen was 4.4, the combined mortality risk factor COVID-19 disease generated from RDW-CV, NLR, MNR and PLT is best as a mortality haematocytometric index.

## 1. Introduction

Coronavirus disease 2019 (COVID-19) is an infectious disease caused by severe acute respiratory syndrome coronavirus 2 (SARS-CoV-2) which was first detected in China, Wuhan in December 2019 and subsequently the WHO announced COVID-19 as a pandemic on March 11, 2020, since the SARS-CoV-2 viral infection has rapidly spread from Wuhan to other provinces in China and around the world. As of 11 March 2020, there have been 118,161,913 confirmed cases and 2,622,143 confirmed deaths [1, 2].

Like the other respiratory coronaviruses SARS-CoV and MERS-CoV; COVID-19 virus enters target cells by using the same primary human host receptor, angiotensin converting enzyme 2 (ACE2). Spike glycoprotein on the surface of the COVID-19 binds to angiotensin-converting enzyme 2 (ACE2) receptor protein located on the host cell membrane and a serine protease TMPRSS211 produced by a host cell facilitates the host cell invasion. COVID-19 virus triggers innate and adaptive antiviral responses, exaggerates immune response leads to respiratory failure and complications which may result in death. Hyperinflammation, endothelial damage, hypercoagulability, renin-angiotensin-aldosterone system (RAAS) imbalance, all play an important role in pathogenesis of COVID-19.

Although more than 85% of COVID-19 patients have a self-limiting illness of mild to moderate symptoms, 15% of patients had severe disease that may deteriorate to multiple organ failure, leading to death. The mortality rate is much higher in elderly patients and in those with pre-existing risk factors, including being male, having an elevated body mass index (BMI), and the presence of underlying co-morbidities such as obesity, diabetes, hypertension, cardiovascular disease, chronic respiratory disease [2–5]. Typically, COVID-19 progresses to a severe form in 10–20% of patients, resulting in the need for hospital admission and/or intensive care unit (ICU) treatment. Therefore, predicting the progression of disease has acquired major clinical significance.

The clinical features of COVID-19 patients, have revealed that many laboratory parameters associated with severity of disease. Particularly, the serum levels of ferritin, D-Dimer, LDH, CRP, troponin I, procalcitonin and an inflammatory cytokine IL-6, have all been proposed to carry prognostic significance. Numerous studies carried out in parallel have also suggested that haemocytometric parameters, including lymphocyte, platelet, neutrophil and monocyte counts appear to correlate with the severity and mortality of COVID-19 cases [3, 4]. Although lymphopenia is a prominent finding in most patients, some studies have reported an increase in the number of neutrophils. In particular, the total number of leukocytes in patients varies between according to lymphocyte suppression or neutrophil increase. Decreased lymphocytes, accompanied by mild thrombocytopenia, are among the most common signs of abnormal CBC in COVID-19 patients. Overall, these findings showed that lymphocyte count retains a specific clinical and biological significance in COVID-19 and lymphopenia is a significant hematological abnormality that negatively affects prognosis. There are some main mechanisms that causes lymphopenia in COVID-19 patients: Direct infections of lymphatic organs, bone marrow, cytokine storm, negative effects of some metabolic products, epigenetic alterations (age, gender, some gene expressions, methylation pattern of some immunoregulatory genes). Neutrophil to lymphocyte ratio (NLR) is a dominant biomarker of systematic inflammation and is commonly used to predict the outcome of bacterial infections, especially those of patients with pneumonia. Apart from bacterial infections, NLR can also provide valuable information about patients’ prognosis in other inflammatory diseases such as cancers, acute coronary syndrome, intracerebral bleeding, polymyositis, and dermatomyositis. It is suggested that in severe cases of COVID-19, the shift of WBCs towards neutrophils rather than lymphocytes occurs, and possibly the calculation of NLR may help the clinician to properly treat patients [6, 7].

The main objective of this study is to identify and evaluate the use of key haemocytometric parameters associated with SARS-CoV-2 infection which may be correlated with the severity of the disease. Although many studies have been conducted investigating the relationship between disease severity-mortality and laboratory parameters, in our study, the course of the disease is more realistically estimated using only routine haematocytometric parameters and the mortality risk factor extracted from these parameters. The prediction that disease prognosis can be followed by routine CBC test follow-up alone has made this study unique. A retrospective analysis was performed on samples gathered from some 97 individuals admitted to Acıbadem Health Care Hospital Group, in order to observe the potential meaning of these parameters, in conjunction with other known factors about diagnosis and treatment, in the clinical guidance of COVID-19 patients in hospital.

## 2. Patients and methods

### 2.1 Patient selection

A total of 97 patients, all diagnosed with COVID-19 and admitted to the Acıbadem Health Care Hospital Group between 15th March, 2020 and 15th June 2020, were included in the study. There were 935 sequential complete blood count (CBC-Diff) measurements (days 0–30) included in the analysis. These data provide a record of the haemocytometric trends over time. Patients with chronic diseases such as diabetes mellitus, hypertension, chronic arterial disease, chronic obstructive pulmonary disease, allergy/asthma and others (representing 71% of COVID-19 patients as a whole) were participated in the study group. However, patients who were pregnant or were suffering from malignancy were not included in the study. The patients’ median age was 56 years (between17-87 years) and 64.9% of the patients were male (Table 1).

**Table 1:** Demographics of COVID-19 patients.

Characteristics	All (n:97)	Non-critical/ Critical survivors (n:82)	Non- Survivors (n:15)	P value
**Age, years median**	56	51,7 ± 14.5	71.7 ± 14.7	<0.0001
**Gender (Female/Male)**	34/63	31/51	3/12	0.133
**Comorbidities**:				
**Diabetes, Hypertension, CVS**	46	35	11	0.0468
**Kidney Disease**	4	2	2	0.111
**Allergy, asthma**	4	4	0	1.000
**Chronic pulmonary disease**	8	6	2	0.605
**Others (autoimm, obesity, thyroid disease)**	7	7	0	0.591
**Any**	28	28	0	1.000

All COVID-19 patients were confirmed by real time reverse transcriptase-polymerase chain reaction (RT-PCR) of pharyngeal and nasal swab specimens and presence of ground-glass opacity (GGO) in the lung, confirmed radiologically.

All participating patients were informed about what the study involved and a signed, voluntary consent form was obtained prior to enrolment. The study ethics approval was granted by the Ethics Board of Acıbadem University, Istanbul, Turkey.

Blood samples were collected in K_2_EDTA-Vacusera plastic tubes and analysed within 4 hours of collection. The leucocyte and sub-parameters, erythrocyte and platelet counts and their indices were analysed, together with the complete laboratory data obtained.

The patient group was subdivided into three major groups: Non-critical (Mild/Moderate), Critical-Survivors (Severe) and Critical-Non-survivors (deaths), following the scheme employed in the COVID-19 directory of the Ministry of Health of the Republic of Turkey [8]. Our non-critical group of patients is a group of patients with mild/moderate pneumonia who require hospitalization that shows the following signs:

- With symptoms such as fever, muscle / joint pain, cough, sore throat, number of breaths <30 / min, Room Air SpO2 (Peripheral capillary oxygen saturation) >90,
- Signs of mild to moderate pneumonia on a lung chart or tomography

Critical patient group is a group of patients with severe pneumonia who require hospitalization and have the following characteristics:

- With symptoms such as fever, muscle / joint pain, cough, sore throat, number of breaths >30 / min, Room Air SpO2<90,
- Patients who showed signs of bilateral widespread pneumonia on a lung chart or tomography [8].

All haemocytometric data were then compared with the data available on clinical severity, symptom duration and days of hospitalisation, and examined up to day 30, with the aim of identifying specific patterns and undertaking a trend analysis for each individual parameter.

### 2.2 Data collection

Data on age, sex and laboratory findings were obtained from the electronic medical records, as were the clinical records. The results of the routine CBC analysis were evaluated without additional blood sampling.

Patient demographical information was collated from surveys the patients had completed on admission to hospital, and patient medical history and other data were obtained clinical examinations data stored in the hospital information system (HIS).

The results of the CBC-Diff undertaken at Acıbadem Labmed Clinical Laboratory were evaluated retrospectively. CBC-Diff parameters results (Sysmex-XN-3000 haematology analyzer, Kobe, Japan) were collated from the laboratory information system (LIS).

### 2.3 Laboratory measurements

All samples were processed by the Sysmex XN-series automated hematology analyser (Sysmex Co., Kobe, Japan). There was strict adherence to the standard operating procedures for this procedure, as follows: standard CBC-Diff parameters and also NEUT-RI (neutrophil reactivity index), NEUT-GI (the neutrophil granularity index), NRBC (nucleated red blood cells), IG (immature granulocytes), RE-LYMPH (reactive lymphocytes), AS-LYMPH (antibody-synthesising lymphocytes) were measured. NLR (the neutrophil to lymphocyte ratio) and MNR (monocyte to neutrophil ratio) were also measured.

Sysmex’s XN-Series analysers permit a quantitative evaluation of the activation status of neutrophils (NEUT-RI, NEUT-GI), IG and activated lymphocytes (RE-LYMP, AS-LYMP). These parameters are determined using fluorescence flow cytometry. A standard blood sample is adequate for the test and the same sample can be used for the CBC-Diff.

The NLR is calculated by dividing the neutrophil count by the lymphocyte count. The MNR is the result of dividing the monocyte count by the neutrophil count.

### 2.4 Statistical analysis

In this research all analyses were performed by using SPSS for Windows, Version 24, (SPSS Inc., Chicago) and Analyse-it for Microsoft Excel (Analyse-it Software, Ltd, The Tannery, 91 Kirkstall Road, Leeds, United Kingdom). The distribution normality of the data was confirmed by the Shapiro Wilk test prior to further analysis. Identifier statistics are provided as mean ± SD and median (Inter-quartile range, IQR). Intergroup comparisons of clinical and hematological parameters were evaluated by ANOVA test with a Bonferroni correction. Receiver operating characteristics (ROC) and the corresponding area under the curve (AUC) analyses for all parameters were fulfilled to permit differentiation in COVID-19 prognosis. Binary Logistic regression via multivariable analysis was used to exploit the risk factors for mortality.

## 3. Results

A total of 97 patients with confirmed COVID-19 infection were grouped into 82 (Mild/ moderate/severe) and 15 non-survived patients to observe and obtain the mortality risk score. Of the 82 patients, 51 (mild/moderate/severe) were males, 31 females, with a median age of 51.7 years. For the 15 non-survived patients, there were 12 males, 3 females, with a median age of 69.2 years.

The haemocytometric parameters where there existed significant differences amongst the 3 groups and between the non-critical, critical-survivors, critical (non-survivors) are presented in Table 2. As the disease progressed, RBC, Hgb, Hct, LYMPH%, MONO%, EO%, PLT, RE-LYMPH%, MNR gradually decreased, whereas MCV, RDW-CV, NEUT%, PDW, MPV, P-LCR, NEUT-RI, NLR gradually increased. The differences among the groups remained significant over time (p <0.05).

**Table 2:** Comparison of haematological parameters with standard deviation between mild, moderate/severe and death groups, and survivor/non-survivor groups in COVID-19 patients.

Parameter	Non-critical (Mild/Moderate) Group 1 (n = 59)	Critical-Survivors (Severe) Group 2 (n = 23)	Critical-Non-survivors (Death) Group 3 (n = 15)	P value
**Age(years)**	49.2 ± 15.1	58.6 ± 16.4 *	69.2 ± 10.6*^,^^&^	<0.001
**Gender (F/M)**	22/37	9/14	3/12	
**RBC (x10^6^/uL)**	4.49 ± 0.64	3.97 ± 0.71 *	3.67 ± 0.65*	0.008
**Hgb (gr/dL)**	12.7 ± 1.8	11.34 ± 2.04*	10.88 ± 1.99*	0.018
**Hct (%)**	37.94 ± 4.8	34.4 ± 5.79*	32.89 ± 6.01*	0.004
**MCV (fL)**	84.97 ± 5.46	86.81 ± 3.7	89.61 ± 4.34*	0.008
**MCH (pg)**	28.41 ± 1.97	28.55 ± 1.18	29.65 ± 1.53	0.124
**MCHC (g/dl)**	33.43 ± 1.19	32.91 ± 1.12	33.12 ± 1.41	0.085
**RDW-CV (%)**	13.29 ± 1.28	14.29 ± 1.35*	15.01 ± 1.9*	0.007
**WBC (ˣ10**^**3**^**/L)**	7.3 ± 4.51	10.92 ± 5.69*	10.84 ± 8.31*	0.005
**NEUT (%)**	65.56 ± 15.09	76.24 ± 12.38*	85.55 ± 7.92*^,^^&^	0.002
**LYMPH (%)**	23.6 (13.1–32.3)	12.65 (7.3–19.5)*	7.6 (5.25–11.55) *^,^^&^	0.001
**MONO (%)**	8.5(6.9–10.6)	7.0 (4.95–9.22)*	5.0 (3.45–7.15) *	0.001
**EO (%)**	0.8 (0.20–2.10)	0.5 (0.10–1.65)	0.2 (0.0–1.10) *	0.015
**BASO (%)**	0.3 (0.2–0.5)	0.3 (0.1–0.4)	0.2 (0.1–0.4)	0.085
**PLT (**^**x**^**10**^**3**^**/μL)**	241 ± 75	219 ± 84	90 ± 70 *^,^^&^	0.001
**PDW (fL)**	11.69 ± 1.89	14.29 ± 1.35*	15.01 ± 1.9*	0.011
**MPV(fL)**	10.26 ± 0.89	10.42 ± 1.36	13.37 ± 2.74*	0.014
**P-LCR (%)**	26.97 ± 7.08	28.08 ± 10.74	32.55 ± 8.23*	0.006
**PCT (%)**	0.27 ± 0.11	0.33 ± 0.15	0.17 ± 0.1*^,^^&^	0.002
**AS-LYMPH (%)**	0 (0–0.5)	0 (0.0.47)	0 (0–0.5)	0.093
**RE-LYMPH (%)**	1.9 (1.0–2.7)	1.2 (0.6–2.0)*	0.9 (0.5–1.3) *^,^^&^	0.003
**NEUT-RI**	49.64 ± 3.88	50.93 ± 3.98	53.77 ± 7.89*	0.014
**NEUT-GI**	151.24 ± 5.4	151.7 ± 5.08	150.78 ± 4.97	0.459
**IG (%)**	0.5 (0.3–1.1)	1.4 (0.8–2.9)*	0.9 (0.8–1.6) *^,^^&^	0.005
**NRBC (%)**	0 (0–0)	0 (0–0.1)	1 (0–0.1) *	0.021
**NLR**	2.67 (1.8–5.1)	6.2(3.2–11.3)*	11.4 (6.9–16.8) *^,^^&^	<0.001
**MNR**	0.139 (0.09–0.189)	0.092 (0.059–0.136)*	0.060 (0.038–0.087) *^,^^&^	<0.001

Abbreviations: F:Female, M:Male, RBC: Red Blood Cell Count, Hgb: Hemoglobin, Hct(%): Haematocrit, MCV: Mean Cell Volume, MCH: Mean Cell Hemoglobin, MCHC: Mean Cell Hemoglobin Concentration, RDW-CV: Red Blood Cell Distribution Width-CV, WBC(%): % of White Blood Cells, NEUT(%): % of Neutrophils, LYMPH (%): % of Lymphocytes, MONO(%): % of Monocytes, EO(%): % of Eosinophils, BASO(%): % of basophils, PLT: Platelet Count, MPV: Mean Platelet Volume, PDW: Platelet distribution width, P-LCR: Platelet-Large Cell Ratio, PCT(%): Plateletcrit, AS-LYMPH(%):percentage of antibody synthesising lymphocyte, RE-LYMPH(%): Percentage of reactive lymphocyte, NEUT-RI: Neutrophil reactivity index, NEUT-GI: Neutrophil granularity index, IG(%): % of immature granulocytes, NRBC(%): % of nucleated red blood cells, NLR: Neutrophil lymphocyte ratio, MNR: Monocyte neutrophil ratio,

*,^&^: compared to critical survivors, critical non-survivors.

All comorbidity conditions for mortality are combined and regression analysis results are included in Table 3. According to age and comorbidity, MNR, Hg, MCV, RDW and PLT were evaluated as independent risk factors for mortality.

**Table 3:** Multivariate logistic regression analysis of laboratory and demographic parameters according to mortality.

					95% C.I.for EXP(B)	
	B	Wald	Sig.	Exp(B)	Lower	Upper
**Gender**	0.11	0.139	0.710	1.114	0.631	1.96
**Comorbidity**	-0.03	0.021	0.886	0.968	0.618	1.52
**NLR**	0.01	1.851	0.174	1.014	0.994	1.03
**MNR**	-64.19	15.855	*<0*.*0001*	0.000	0.000	0.00
**MONO %**	0.53	5.621	0.018	1.692	1.095	2.61
**WBC**	-0.04	6.931	0.008	0.957	0.927	0.99
**HGB**	-0.01	0.066	0.797	0.985	0.881	1.10
**MCV**	0.16	35.658	*<0*.*0001*	1.172	1.112	1.23
**RDW**	0.29	15.984	*<0*.*0001*	1.331	1.157	1.53
**PLT**	-0.01	62.902	*<0*.*0001*	0.990	0.988	0.99
**Constant**	-15.27	28.094	*<0*.*0001*	0.000		

**Fig 1:**
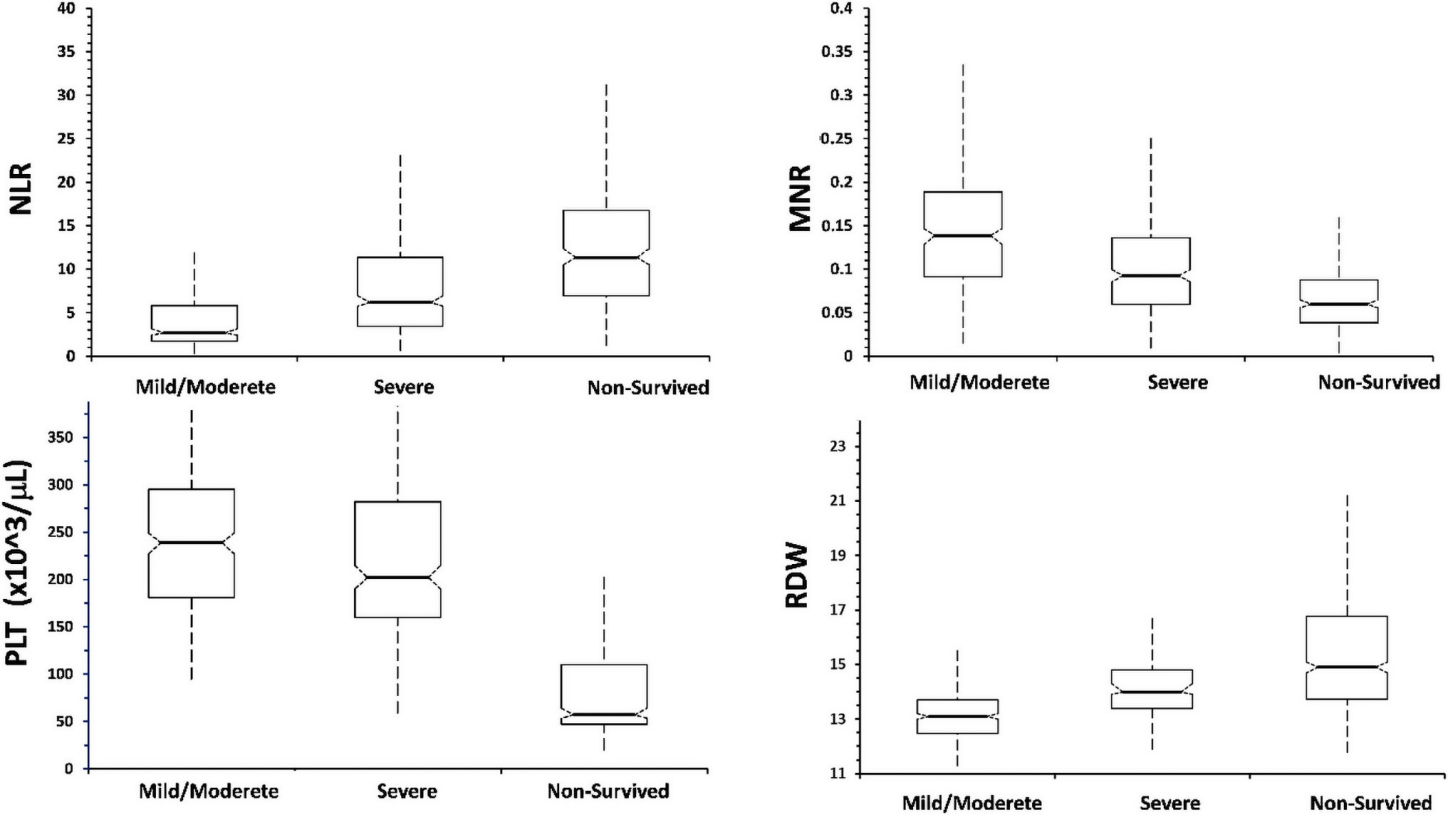
Comparison of hematological parameters with standard deviation between the types of COVID-19 patients.

A grouped box plot for NLR, MNR, PLT and RDW is shown in Fig 1. Each parameter gradually increased or decreased as the disease progressed. Significant differences were found between the 3 groups in terms of NLR, MNR, RDW and PLT and prognostic assessments were performed for each.

The prognostic parameters related to the severity of disease in the three groups are in Table 4. The sensitivity and specificity values were 58.8% and 78.9% for MNR, with a cut-off value of 0.08, or 69.5% and 72.1% for MNR with a cut-off value of 0.1.

**Table 4:** Prognostic evaluation of the best parameters to indicate severity of disease.

Parameters	Cut-offs	TP proportion (Sensitivity)	TN proportion (Specificity)	PPV	NPV	Accuracy	Likelihood ratio (+)	Likelihood ratio (-)	Odds ratio
**NEUT(%)**	72.3	0.790	0.670	0.826	0.618	0.750	2.40	0.31	7.666
**LYMPH(%)**	15.3	0.752	0.713	0.840	0.590	0.739	2.62	0.35	7.526
**NLR**	3.10	0.870	0.541	0.789	0.678	0.759	1.90	0.24	7.896
**NLR**	4.40	0.785	0.682	0.830	0.616	0.750	2.46	0.32	7.801
**MNR**	0.08	0.588	0.789	0.846	0.492	0.655	2.78	0.52	5.322
**MNR**	0.10	0.695	0.721	0.831	0.545	0.704	2.49	0.42	5.885
**MONO (%)**	7.1	0.626	0.738	0.825	0.500	0.664	2.39	0.51	4.720
**WBC**	7.18	0.676	0.670	0.802	0.512	0.674	2.05	0.48	4.248
**Hgb**	11.4	0.641	0.786	0.855	0.526	0.690	3.00	0.46	6.567
**MCV**	86.7	0.646	0.665	0.792	0.488	0.652	1.93	0.53	3.615
**RDW-CV**	13.7	0.677	0.768	0.855	0.540	0.707	2.92	0.42	6.945
**PLT**	160	0.654	0.818	0.366	0.43	0.92	3.58	0.42	8.454

The sensitivity and specificity values were 87% and 54.1% for NLR, when the cut-off value chosen was 3.1, and 78.5% and 68.2% when the cut-off value was 4.4, respectively. Although these results are statistically significant, none of these parameters are associated with high clinical validity (+Likelihood Ratio (LR) > 10 and–LR < 0.1) [2].

**Fig 2:**
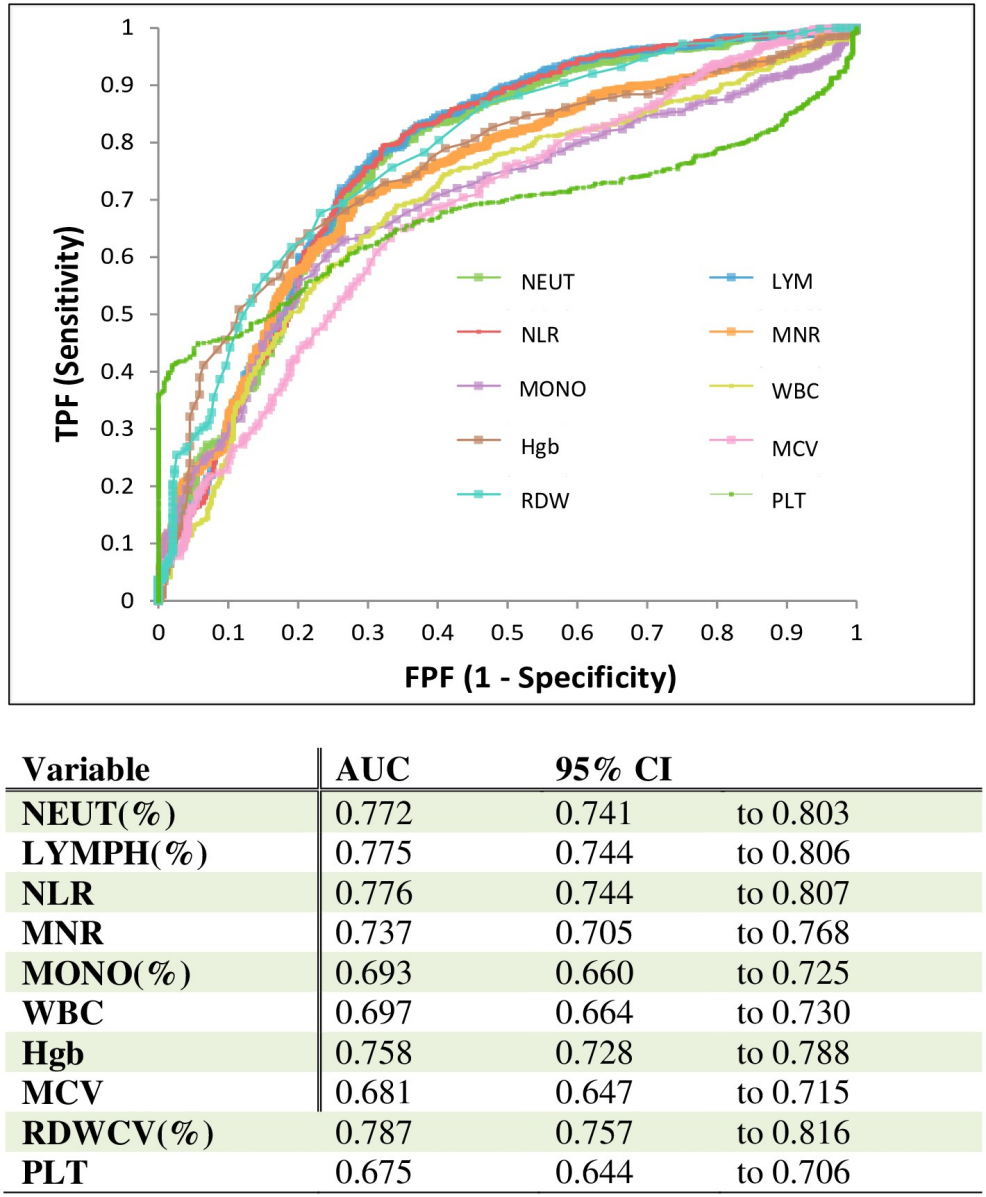
ROC analysis of hematological parameters in the diagnosis of severe COVID-19 patients.

The ROC curve used to analyse the diagnostic and prognostic value of each parameter of the CBC-Diff in distinguishing the level of severity is shown in Fig 2. Amongst all the haemocytometric parameters RDW-CV, Neut %, Lymph % and NLR yielded the best area under the ROC (receiver operating characteristic) curve (0.787, 0.772, 0.775, 0.776, respectively) for predicting severity.

**Fig 3:**
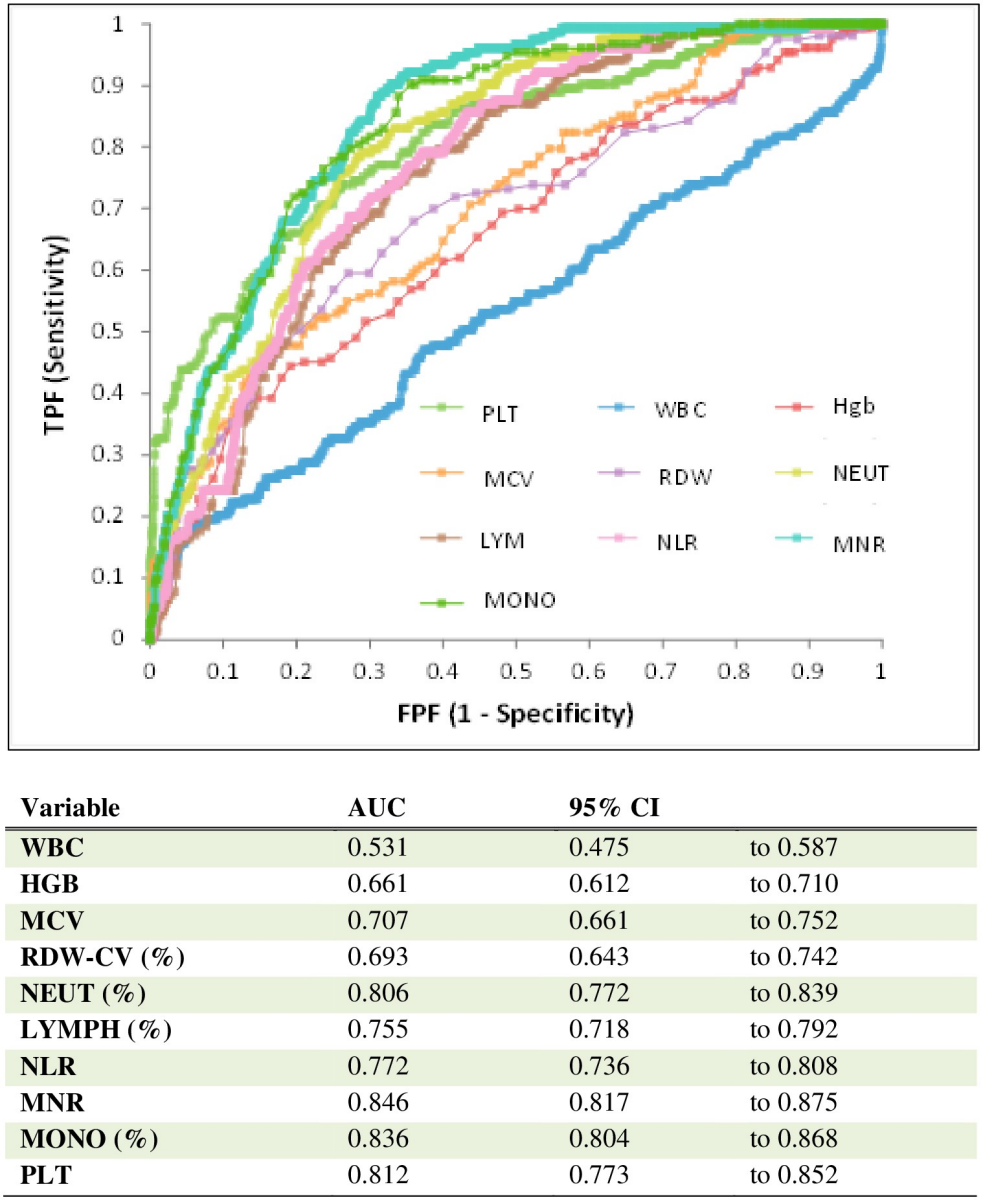
ROC analysis using combined parameters for the assessment of mortality risk in COVID-19 cases.

MNR yielded the best AUC (0.846) for predicting mortality from COVID-19. Furthermore, the values for AUC obtained with Mono %, PLT %, Neut % and NLR were 0.836, 0.812, 0.806 and 0.772, respectively. Thus these parameters were also found to be significant biomarkers for predicting mortality (Fig 3).

The prognostic parameters used to calculate mortality risk from the disease in assessments of the two groups are listed in Table 5. The sensitivity and specificity values were 88.9% and 68.4% for MNR, when the cut-off value was 0.08, and 92.8% and 61.4% for MNR when the cut-off value was 0.095.

**Table 5:** Prognostic evaluation of the best parameters to show mortality from disease.

Parameter	Cutoff	TP proportion (Sensitivity)	TN proportion (Specificity)	Accuracy	PPV	NPV	Likelihood ratio (+)	Likelihood ratio (-)	Odds ratio
**NLR**	3.101	0.961	0.380	0.483	0.251	0.978	1.55	0.10	15.0
**NLR**	5.233	0.856	0.568	0.619	0.300	0.948	1.98	0.25	7.82
**MNR**	0.084	0.889	0.684	0.720	0.378	0.966	2.81	0.16	17.2
**MNR**	0.095	0.928	0.614	0.670	0.342	0.975	2.41	0.12	20.5
**EO (%)**	0.200	0.608	0.734	0.712	0.331	0.897	1.99	0.53	4.28
**MONO (%)**	6.900	0.902	0.638	0.685	0.350	0.968	2.44	0.15	16.2
**LYMPH(%)**	11.500	0.739	0.671	0.683	0.324	0.923	2.23	0.39	5.75
**NEUT(%)**	81.400	0.791	0.710	0.725	0.371	0.940	2.73	0.29	9.27
**WBC**	19.320	0.157	0.959	0.816	0.453	0.840	3.67	0.88	4.35
**Hgb**	10.2	0.444	0.808	0.257	0.556	0.192	2.31	0.68	3.36
**MCV**	90.0	0.477	0.840	0.224	0.523	0.160	2.98	0.62	4.79
**RDW-CV**	14.0	0.647	0.666	0.337	0.353	0.334	1.94	0.53	3.66
**PLT**	142	0.411	0.980	0.976	0.458	0.603	20.9	0.60	34.7

For NLR, the sensitivity and specificity values were 96.1% and 38% when the cut-off value chosen was 3.1, and 85.6% and 56.8% when the cut-off was 5.23. When their diagnostic and prognostic characteristics were examined, it was determined that they were not sufficient to be used to both rule-in and rule-out mortality. (+LR was 2.81, 2.41 for MNR and–LR was 0.16, 0.12 for MNR) (Table 5).

**Fig 4:**
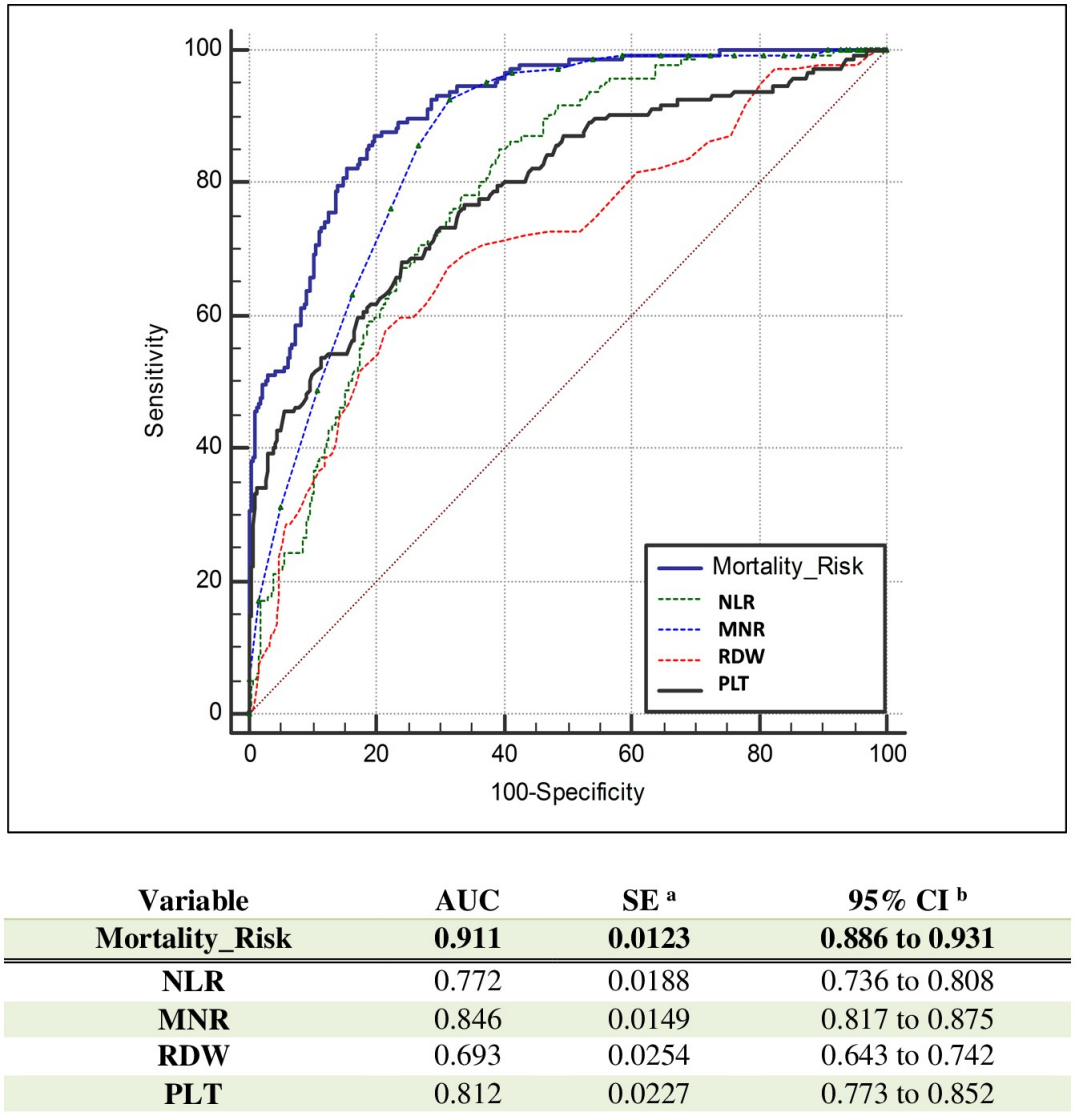
ROC curves of mortality risk and NLR, MNR, RDW, PLT.

When all four parameters (MNR, NLR, RDW, PLT) were combined, the AUC was 0.911, LR+ was 9.87 and the optimal value in mortality risk assessment was obtained. (Fig 4 and Table 6) In assessing the risk of mortality, the sensitivity was 61.6%, with a specificity of 93.8%. Even when the blood parameters as a whole are examined, the adequacy of PPV, NPV, LR+ and LR- values for diagnostic evaluation is limited.

**Table 6:** Prognostic evaluation of combined parameters for mortality from the disease.

Parameter	TP proportion (Sensitivity)	TN proportion (Specificity)	Accuracy	PPV	NPV	Likelihood ratio (+)	Likelihood ratio (-)	Odds ratio
**Mortality risk**	0.616	0.938	0.848	0.796	0.861	9.873	0.410	24.1

**Fig 5:**
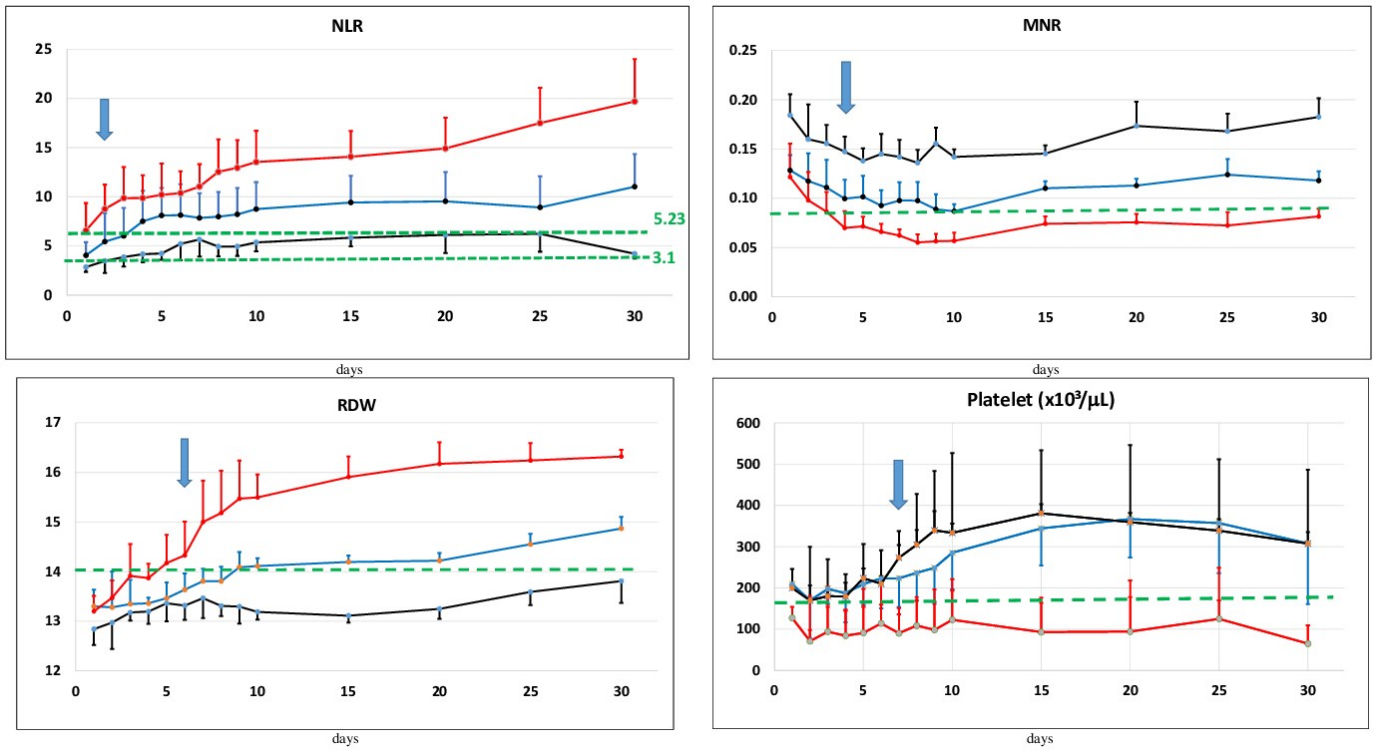
Trend graphics of time-related mortality risk factor parameters. Abbreviations: Black line: Non-critical survivors, Blue line: Critical-survivors, Red line: Non-survivors. Trends of CBC-Diff parameters over 30 Critical-non-survivors days of hospitalisation in mild/moderate, severe, non-survivor patients. 30 days of hospitalisation refers to Day 0 and the next days after admission. The reference range is displayed by the green dotted horizontal line. NLR: Neutrophil lymphocyte ratio, MNR: Monocyte neutrophil ratio, RDW-CV: Red Blood Cell Distribution Width-CV, PLT: Platelet (^x^10^3^).

In the present study, MNR and NLR were the best time-related markers over the course of the disease, distinguishing non-critical patient group from non-survivor patient group after the 2th-4th day of admission, PLT was the best time-related parameter after the 7th day of admission and RDW was the best time-related parameter after the 6th day of admission (Fig 5).

**Fig 6:**
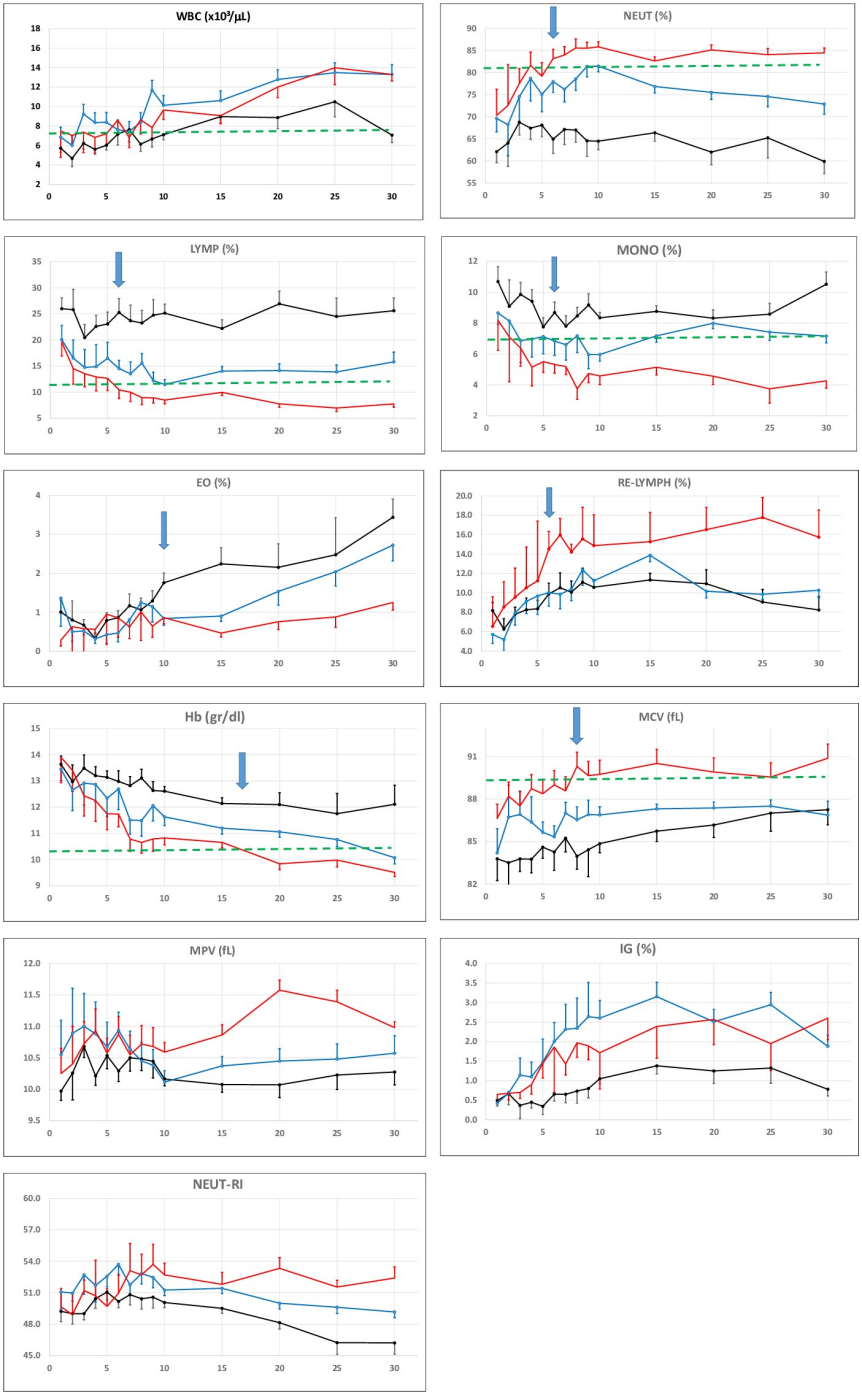
Trend graphics of time-related CBC-Diff parameters. Abbreviations: Black line: Non-critical survivors, Blue line: Critical-survivors, Red line: Non-survivors. Trends of CBC parameters over 30 days of hospitalisation in mild/moderate, severe, non-survivor patients. 30 days of hospitalisation refers to Day 0 and the next days after admission. The dotted horizontal line: Limit of reference value. WBC (^x^10^3^): of White Blood Cells, NEUT (%): % of Neutrophils, LYMPH (%): % of Lymphocytes, MONO (%): % of Monocytes, EO (%): % of Eosinophils, RE-LYMPH (%): % of Reactive lymphocyte, Hct (%): Haematocrit, Hgb: Haemoglobin (g/dl), MCV: Mean Cell Volume (fL), IG (%): % of immature granulocytes, MPV: Mean Platelet Volume (fL), NEUT-RI: Neutrophil Reactivity Index.

When we make trend analysis of parameters other than mortality risk factor components; we found that Neut%, Lymph %, Re-Lymph% and MCV were distinctive especially after 7th day in the critical group (Fig 6). When we evaluate all parameters together; we noticed a divergence for all groups in all parameters after the 10th day (Figs 5 and 6).

## 4. Discussion

From the beginning of COVID-19 outbreak in December 2019, as the SARS-CoV-2 virus spread globally, establishing an effective treatment for critical and fatal patients has depended on early warning progression signs and diagnosis of disease, which remain the hint for reducing overall mortality of the disease.

COVID-19 does not exhibit severe features in the beginning stage, but those patients who become critically ill deteriorate within 10 days of developing the illness and enter a stage of severe pneumonia, ARDS. Patients with COVID-19 who enter a critical condition or die are mostly elderly and have comorbidities [5, 9].

Therefore, the CBC-Diff as a convenient and effective measurement in clinical practice, capable of providing rich information about haemacytometric parameters can be used to evaluate the severity of the disease, monitor the treatment process and the mortality risk and provide information for targeted or preventive medication [1].

The main objective of this study was to evaluate the haemocytometric parameters of COVID-19 patients with the different disease degree and how the results changed after disease onset to identify key indicators of disease progression and severity and to obtain a basis for diagnosis and treatment for clinicians.

When we examined certain haemocytometric parameters in the critical-severe and non-survivor groups, we noted how these parameters were significantly raised compared with those in the non-critical (mild/moderate) group (P<0.05). Meanwhile, RBC, Hgb, Hct (%), Lymph (%), Mono (%), Eo (%), PLT, Re-Lymph (%), MNR in the critical-severe and non-survivor groups were noted to be significantly lower than those in the mild/moderate group (P<0.05). These findings are consistent with the results reported by Wang *et al*., Chen *et al*. [1, 5, 9].

Most studies indicate that increased NLR and lymphopenia are the most consecutively abnormal haemocytometric parameters [10, 11]. Our present study revealed that elevated NLR alongside decreased MNR, lymphocyte and platelet levels are more likely to exist in critical patients, compared with those non-critical patients.

The results of our study largely replicate those reported above, however we found in addition that MNR has a diagnostic value in differentiating non-critical COVID-19 patients from critical patients.

There are limited studies were performed on the variation of time-dependent CBC parameters. A recent multi-centrebased study by Linssen at al., CBC changes with new parameters such as As-Lymph, Re-Lymph, Neut-RI and IG were retrospectively analysed for 14 days of hospitalisation in the larger data group. Although our study is a single-centre study with a small sample size it is a unique study in terms of determining the mortality risk by monitoring all CBC parameters for 30 days of hospitalisation [12].

In this study they found that lymphopenia remained stable for 7 and 10 days respectively in both groups, neutrophil count was normal in the non-critical group, whereas values continue to increase over time in the critical group, monocyte and platelet counts were showed upward trend a gradual increase from about day 10 onwards in critical patients, the NLR increases in both the critical and non-critical patients group whilst our study concluded that the NLR value was higher in the critical group than non-critical group from day 2 as an early discrimination power [12].

In contrast to this study, we demonstrated that the lymphocyte from day 5, platelet and monocyte count from day 10 were decreased, Re-Lymph was increased from day 5 in critical patient group with significantly difference between the survivor and non-survivor group (Fig 5), however the time dependent variation of the other new parameters such as As-Lymph, Neut-RI and IG were not sufficient to discriminate patients based on disease severity. In parallel to their study, there was a gradual decline in Hg while MCV becoming increase with differences between the non-critical and critical groups from day 10 (Fig 6).

An increased NLR is identified as an increased neutrophil count in conjunction with a decreased lymphocyte count. Lymphocytes are the important part of the inflammatory response and neutrophils are important cells in the innate immune mechanism. Therefore, the imbalance of the inflammatory stage may indicate a high NLR and is a potential marker of clinical risk assessment and outcome in many infections as a simple and standalone assay [4, 11, 13].

Several studies have described that critical-severe COVID-19 cases were likely to have higher neutrophile but lower lymphocyte counts compared with non-critical (mild/moderate) patients as follows.

A NLR and an age-based risk prediction model was offered by Liu *et al*. to advance risk formation and clinical control. In this study, the incidence of critical illness was only 9.1% in patients aged 50 years with NLR <3.13, whereas 50% of patients aged 50 years with NLR over 3.13 developed critical illness [9, 10].

In the study by Zhang *et al*., just as an increased NLR in high-risk groups, some 94% of the 82 deceased patients with COVID-19 were reported to have a NLR >5.0 [13].

Yan et al. showed that in multivariate logistic regression analysis, NLR bigger than 11.75 was significantly correlated with all hospital mortality (odds ratio = 44.3; 95%) [11].

Wang *et al*. concluded that an elevated NLR upon admission to hospital was an independent predictive marker of severe pneumonia in COVID-19 cases [14, 15].

The study by Pirsalei et al. shows that a rate of NLR greater than 6.5 can negatively affect the prognosis of the disease, and that rate higher than 9 can strongly result in death [16].

Our results stated that the NLR yielded a relatively high AUC (0.776, 95% CI: 0.741–0.803) when the ROC was used to predict severity of the disease with a cut-off set to 3.1(sensitivity 87%, specificity 54.1%). When the cut off value chosen is 4.4, sensitivity decreases to 78.5% but specificity rises to 68.2% (Table 4). In regard to the prediction of mortality, when the cut-off was 3.1, sensitivity was 96.1% and specificity 38%, whereas when the cut-off chosen was 5.23, the sensitivity decreased to 85.6% whilst specificity rose to 56.8% (Table 5). These parameters are insufficient to both rule-in and rule-out likely severity and mortality.

All of these clinical studies have shown that NLR is a predictor of mortality in the critical patient group with COVID-19 and dysregulation of the immune response is one of the hallmarks of severe SARSCoV-2 infection, with lower lymphocytes counts and an increased NLR.

Coinciding with these studies, our results support that a high calculated NLR, especially NLR > 5.23 is a good predictive factor for poor outcome in COVID-19 patients.

Macrophages and monocytes are important actors in innate and adaptive immune response against microbial and viral pathogens through the production of cytokines, phagocytic activity, and activation of lymphocytes [17]. Many studies have concluded that severe COVID-19 pneumonia is related with cytokine storming and over-activation of monocytes and macrophages.

A number of studies denote an increased monocytes count, however some studies, Sanchez-Cerillo *et al*., found a substantial decrease in monocytes count in COVID-19 patients [18, 19]. In a retrospective study, Pirsalehi et al reviewed the results of WBC and monocyte counts of 1320 COVID-19 patients (243 of whom had severe disease) both on admission and within a 7-day follow -up. They found that both the number of monocytes and the percentage of monocytosis were higher in the severe patients than non-severe patients [20]. Shulte-Schrepping *et al*., recently reported depletion of all nonclassical monocytes in COVID-19. In Padgett et al. study one of the subsets of nonclassical monocytes (nMo2) were nearly absent in both moderate and severe COVID-19 patients [21, 22].

We demonstrated that there is an important variation between survivor and non-survivor COVID-19 patients in terms of monocyte count and we indicated that the MNR was lower in critical group than non-critical group as well. Monocyte (%) reduction may also be associated with selective recruitment of monocytes in the lungs during the development of ARDS. The other possibility, COVID-19 patients are more susceptible to microbial superinfections during long and ICU treatment and this may contribute to changes in inflammation, migration, and homeostasis of myeloid cells, especially in the group whose disease undergoing severe progression [18]. In view of the dynamic changes in the monocyte compartment after the onset of the disease in SARS-CoV-2 infection in relation to the severity, it is important to analysis the monocyte parameter at sequential time intervals in duration of disease.

In our study the MNR yielded a relatively high AUC (0.737 95% CI:0.705–0.768) for predicting severity when the cut-off is set to 0.08, but the sensitivity was just 58.8%, whilst specificity was 78.9%. With the cut-off set to 0.1, the sensitivity increases to 69.5% and specificity decreases to 72.1% (Table 4). For predicting mortality, with cut-off set to 0.084, the sensitivity was 88.9% and specificity was 68.4%, however, with a cut-off value of 0.095 the sensitivity increased to 92.8%, with a decrease in specificity to 61.4%. These values are not satisfactory for either rule-in out rule-out of particular levels of severity or possible mortality.

In this study, Neut % increases significantly with the severity of the disease, and Mono % decreases significantly in correlation with the severity of the disease and also the study found that MNR and NLR were the best time-related markers over the course of the disease for early prediction of poor outcome and mortality risk. Only a few limited studies were conducted considering decreased mono% count and MNR, we suppose that in addition to NLR increase, MNR is significantly reduced in patients with pneumonia [4, 9, 23, 24].

According to the study conducted by Fang *et al*., the ratio of monocytes: neutrophils can be used to determine the severity of sepsis [25]. Téllez *et al*, also reported that NMR and LNR are accurate predictors of in-hospital mortality at admission in patients with severe Covid-19 [26].

Therefore it is possible to assume that NLR and MNR may be related to hyperinflammation and poor outcome in severe COVID-19 patients, and may reflect an imbalance between immune cells. For this reason, MNR and NLR not only suggest a marker of severity and mortality for COVID-19, but also display prospective treatment details aimed at reducing neutrophil over-activation and elevating lymphocyte and monocyte count by immunomodulatory therapy could be a therapeutic option.

Platelets or thrombocytes are involved not only in terms of hemostasis, but also in the mechanisms of inflammation and host defense. Many studies have indicated that thrombocytopenia is an ordinary symptom in COVID-19 patients and related with an elevated mortality risk.

Several mechanisms of COVID-19 associated thrombocytopenia has been suggested: Bone marrow and megakaryocyte suppression due to Inflammatory cytokines, direct viral infection, reduced thrombopoietin can cause thrombocytopenia by destroying progenitors in the bone marrow and reducing platelet. The lung damage of COVID-19 patients causes thrombocytopenia since platelets are released from mature megakaryocytes residing in the lung, structural change or increased pulmonary endothelial damage may also provoke destruction of platelets. Direct PLT-virus interaction associated with formation of PLT-leukocyte aggregates, in addition to PLT activation by increased thrombin generation and consumptive coagulopathy can cause PLT activation and subsequent clearence by reticulo-endothelial system and this way can be one of the possible mechanisms of COVID-19-associated thrombositopenia. [26–28]

In a retrospective single-centered study by Yang *et al*.; A total of 1476 patients, consist of 1238 (83.9%) survivors and 238 (16.1%) non-survivors, were included in the study. The sequential changes in platelet counts are analysed among all patients with COVID-19 in the first 3 weeks after admission. They found that non-survivors had significantly lower platelet counts than survivors (79 [43–129] versus 203 [155 −257], P < .001) [27].

Similarly another study by Liu *et al*., revealed that platelet count as an independent risk factor associated with in-hospital mortality and reported that increment of per 50x10^9^/L in platelets was associated with 40% decrease in mortality among the 383 COVID-19 patients [29].

Bashas et al.’s meta-analysis of 19 studies, totaling 3383 COVID-19 patients with 744 (21.9%) severe cases demonstrated the prognostic value of platelet count in this infection that low platelet count is associated with increased risk of severe disease with the pooled mean difference of -21.5 (%95 CI:-31.57, -11.43) and thus proposed that thrombocytopenic COVID-19 patients will experience disease with a higher risk of adverse outcome. They revealed that non-severe cases have a significantly higher number of platelets and showed that the probability of the emergence of thrombocytopenia is significantly higher in the severe cases with the pooled mean difference of -21.5 (%95 CI: -31.57, -11.43) [30]. Bashash *et al*., also reported that there is publication bias, showing overestimation in studies with lower sample size and incomplete information of sampling time with using asymmetrical form of funnel plot [30]. In our study, we believe that we excluded these problems by dynamic platelet monitoring, excluding underlying medical conditions as much as possible, and using similar sampling times.

Although the most of recent studies revealed a decreasing of platelet level in critical patient group with COVID-19 infection, none of these studies has investigated the time-related changes of platelets and compared in different clinical stage of patients infected with SARS-CoV-2.

In agreement with these studies, we found that thrombocytopenia had a close relationship with the severity of the disease, the critical-severe patients had a lower PLT than non-critical patients. Additionally, those who did not survive group had a much lower PLT than those who critical-survived. The dynamic variation of platelets over 7 days after admission were negatively correlated with the prognosis. Considering the various suggested mechanisms, we conclude that this is an indicator of the severity of the disease. Due to the relationship between PLT and increased risk of mortality, PLT can be followed up as a prognostic hematologic marker during hospitalisation.

The coefficient of variation of RBC distribution width (RDW-CV) is a quantitative measure of the variance in red blood cell size as part of routine parameters of CBC measured by automated haematology counters in clinical laboratories. A higher RDW level indicates abnormal variation in individual red blood cell size, termed anisocytosis, when observed in a blood smear microscopically. An elevated RDW implicates an increased rate of red blood cell (RBC) destruction, dysfunctional erythropoiesis and or shortened RBC lifespan [31, 32].

Although the specific mechanism for the between RDW elevation and adverse outcomes in COVID-19 infection has not yet been established, may be explained by the following potential mechanisms taken together with the recent numerous reports: First, many studies have suggested that COVID-19 infection was associated with an increase in the release and production of white blood cell counts and platelets from the bone marrow. The stimulation to the bone marrow may also cause the dysfunctional erythropoiesis, resulting in shortened RBC lifespan and premature release of RBCs from the bone marrow and subsequently elevated RDW levels. RDW may increase due to in the presence of increased oxidative stress associated with inflammation. Secondly, the overproduction of inflammatory cytokines affect hematopoiesis RBC-lifespan and variability of its size, iron metabolism, hematopoietic response to erythropoietin which eventually lead to the RDW elevation. Another possible mechanism SARS-CoV-2 spike protein binds to the CD147 receptor that plays a role for mediating SARS-CoV-2 invasion and diffusion among other cells; the CD147 receptor is also determinative of the blood group system. The blockage of CD147 by spike protein leads to the elective trapping of RBCs in the spleen and which eventually RDW elevation [33].

In the meta-analysis by Lee et. al. that includes a total of 14,866 subjects from 10 studies was shown that elevated RDW levels were associated with poor outcomes in COVID-19 patients (mean differences = 0.72; 95% CI = 0.47–0.97; I 2 = 89.51%) and non-survived patients had higher levels of RDW compared to patients who survived from SARS-CoV-2 infection (mean differences = 0.93; 95% CI = 0.63–1.23; I 2 = 85.58%). In this meta-analysis, higher levels of RDW were associated with poor outcomes among COVID-19 patients [34].

In a study by Brody H. et al, an elevated RDW-CV (>14.5%) at admission and increasing during hospitalization for SARS-CoV-2 infection were associated with an increase in mortality risk from (11%-31%) in a cohort of 1641 patients treated at a large academic adjustment for patient age, race, ethnicity but the timing of disease onset was not available for this cohort because the time of initial infection was unavailable. These results are not specific to any disease progression time points RDW change in survivors and non-survivors of SARS-CoV-2 infection [35].

Our results showed that the RDW-CV (%) was the most valuable time-related red blood cell parameter where a cutoff value of 13.7 demonstrated a sensitivity of 75.7% and specificity of 81.6% with an area under the ROC curve of 0.787 (95% CI: 0.757–0.816) when used to assess severity. (Fig 2). This study also concluded that RBC, Hg, Hct (red blood cell parameters) significantly decreased in the critical (severe and non survivors) group, while the critical patient group (non-survivor and severe) had a significantly increased RDW-CV (%) as compared to non-critical (mild/moderate) group.

Although RDW appears to be non-specific parameter of the illness that provides general quantitative risk estimation, when used in conjunction with other parameters (MNR, NLR, PLT) to distinguish mortality of COVID-19 patients, it may be particularly useful for COVID-19 prognosis [31, 32].

In this study, after a best fit analysis, the combined parameter of NLR, MNR, RDW-CV and PLT was observed as the best indicator of raised mortality risk in COVID-19 patients. The AUC reached 0.911 while the diagnostic sensitivity rose to 61.6%, and specificity reached 93.8% (Table 6). The initial AUC value of the prognostic score developed by Lessen et al is 0.753 (95% CI 0.723–0.781), while on the 3rd day it rises to 0.875 (95% CI 0.806–0.926), whilst our study was found that MNR, NLR, PLT and RDW were important parameters, and the AUC value of the Mortality Risk measurement created by their combination was quite high (AUC = 0.911, 95% CI, 0886–0.931) [12]. The combined parameter (mortality haematocytometric index.) can help clinicians anticipate the classification of patients and take effective treatment precautions in advance.

This study has limitations, such as insufficient sample size and a single-center retrospective study. Therefore, the specific application of our findings must be verified by larger studies involving multicenter study.

## 5. Conclusion

This study concluded that the mortality risk factor consisting of PLT, NLR, MNR and RDW-CV can be used as an indicator to distinguish critical patients from non-critical COVID-19 patients and to give information on the risk of mortality.

Even when all the whole blood parameters were examined, although the adequacy of performance characteristics were limited for diagnostic evaluation. It is assumed that disease prognosis and mortality can be estimated when using only the mortility haematocytometric index (Table 6) created using CBC parameters within routine laboratory tests. This parameter may be helpful for providing an insight into when early intervention is required [36].

## Supporting information

S1 Dataset (RAR)
